# A case of Meigs’ syndrome with preceding pericardial effusion in advance of pleural effusion

**DOI:** 10.1186/s12890-016-0241-1

**Published:** 2016-05-10

**Authors:** Kenichi Okuda, Satoshi Noguchi, Osamu Narumoto, Masako Ikemura, Yasuhiro Yamauchi, Goh Tanaka, Daiya Takai, Masashi Fukayama, Takahide Nagase

**Affiliations:** Department of Respiratory Medicine, Graduate School of Medicine, The University of Tokyo, 7-3-1 Hongo, Bunkyo-ku, Tokyo 113-8655 Japan; Department of Pathology, Graduate School of Medicine, The University of Tokyo, Bunkyo-ku, Japan

**Keywords:** Meigs’ syndrome, Ovarian fibroma, Pericardial effusion, Pleural fluid

## Abstract

**Background:**

Meigs’ syndrome is defined as the presence of a benign ovarian tumor with pleural effusion and ascites that resolve after removal of the tumor. The pathogenesis of the production of ascites and pleural effusion in this syndrome remains unknown. Aside from pleural effusion and ascites, pericardial effusion is rarely observed in Meigs’ syndrome. Here, we report the first case of Meigs’ syndrome with preceding pericardial effusion in advance of pleural effusion.

**Case presentation:**

An 84-year-old Japanese non-smoking woman with a history of lung cancer, treated by surgery, was admitted due to gradual worsening of dyspnea that had occurred over the previous month. She had asymptomatic and unchanging pericardial effusion and a pelvic mass, which had been detected 3 and 11 years previously, respectively. The patient was radiologically followed-up without the need for treatment. Two months before admission, the patient underwent a right upper lobectomy for localized lung adenocarcinoma and intraoperative pericardial fenestration confirmed that the pericardial effusion was not malignant. However, she began to experience dyspnea on exertion leading to admission. A chest, abdomen, and pelvis computed tomography scan confirmed the presence of right-sided pleural and pericardial effusion and ascites with a left ovarian mass. Repeated thoracentesis produced cultures that were negative for any microorganism and no malignant cells were detected in the pleural effusions. Pleural fluid accumulation persisted despite a tube thoracostomy for pleural effusion drainage. With a suspicion of Meigs’ syndrome, the patient underwent surgical resection of the ovarian mass and histopathological examination of the resected mass showed ovarian fibroma. Pleural and pericardial effusion as well as ascites resolved after tumor resection, confirming a diagnosis of Meigs’ syndrome. This clinical course suggests a strong association between pericardial effusion and ovarian fibroma, as well as pleural and peritoneal fluid.

**Conclusions:**

In female patients with unexplained pericardial effusion and an ovarian tumor, clinicians should consider the possibility of Meigs’ syndrome. Although a malignant disease should be suspected in all patients with undiagnosed pleural and/or pericardial effusion, Meigs’ syndrome is curable by tumor resection and should be differentiated from malignancy.

## Background

Meigs’ syndrome is rare and typically consists of a benign ovarian fibroma with ascites and pleural effusion that resolve after tumor removal [1]. Ovarian fibromas account for approximately 3 % of all ovarian tumors and Meigs’ syndrome occurs in 1–10 % of these cases [2]. Since Meigs and Cass (1937) reported a case series of patients who had ascites and pleural effusion associated with benign ovarian fibroma [3], this entity has drawn widespread attention from the medical profession around the world. However, the pathogenesis of the formation of the ascites and pleural effusion in Meigs’ syndrome is unknown. It has been hypothesized that the fluid accumulation in the peritoneal cavity may result from an imbalance between fluid production and lymphatic drainage in association with the tumor, and the pleural effusion arises secondary to ascites through passage in the diaphragm [1]. An alternative hypothesis is that substances like vascular endothelial growth factor (VEGF), which increase capillary permeability, and some inflammatory cytokines are possible etiologies of ascites and hydrothorax formation [4]. Although pericardial effusion is a coelomic fluid, as are pleural effusion and ascites, it rarely occurs in Meigs’ syndrome. Very few cases with such a condition have been reported in which pericardial effusion was simultaneously detected with other findings [5–7]. We describe the first case of Meigs’ syndrome with preceding pericardial effusion and discuss the association between ovarian fibromas and pericardial effusion.

## Case presentation

**Fig. 1:**
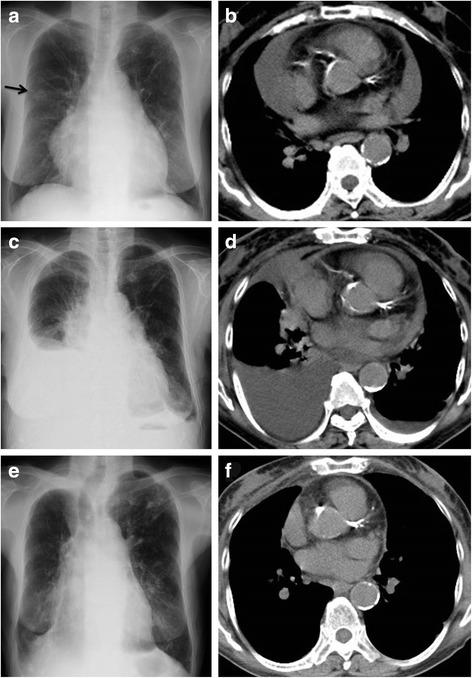
Representative photographs during the clinical course. Chest X-ray and computed tomography (CT) scan 4 months before admission, which was 2 months before the lung cancer operation, revealed cardiac enlargement with a nodular shadow (arrow) in the right lung field (**a**) and a large volume of pericardial effusion (**b**). On admission, a chest X-ray showed the right pleural effusion as well as blunting of the left costophrenic angle (**c**), and a chest CT scan showed that the pericardial effusion had decreased compared with that before the lung operation (**d**). No relapse of the pleural and pericardial effusion was confirmed by chest X-ray or a CT scan 8 months after removal of the fibroma (**e**, **f**)

**Fig. 2:**
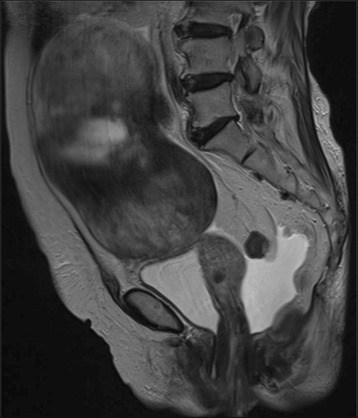
Magnetic resonance imaging of the pelvis before surgery for lung cancer. An abdominal mass with low signal intensity on T2-weighted images and ascites was observed

An 84-year-old Japanese woman with a history of lung cancer treated by surgery was admitted due to gradual worsening dyspnea over the previous month. She had had asymptomatic and unchanging pericardial effusion and a pelvic mass diagnosed 3 and 11 years previously, respectively, and had been followed-up without the need for treatment. The chest radiography and magnetic resonance imaging of the pelvis 4 months before admission are shown in Fig. 1a and Fig. 2, respectively. The chest X-ray showed a nodular shadow in the right lung field (Fig. 1a), as well as unchanging pericardial effusion volume revealed by a chest computed tomography (CT) scan (Fig. 1b). Transbronchial biopsies of the lung nodule were performed, and the pathological specimen disclosed adenocarcinoma. Then, she underwent a right upper lobectomy for localized lung adenocarcinoma with pericardial fenestration, which confirmed that the pericardial effusion included abundant lymphocytes and did not contain any malignant cells. She progressed favorably after the operation and was discharged. However, she began to experience dyspnea on exertion leading to admission. On admission, she was not suffering from fever, night sweats, chest discomfort, or abdominal pain. She had a prior history of pulmonary tuberculosis treated with chemotherapy 20 years before and was treated for diabetes mellitus and hypertension in the hospital. She had no history of smoking, illicit drug use, recent travel abroad, or asbestos exposure.

The patient was conscious and afebrile, with a pulse of 108 beats/min, blood pressure of 148/50 mmHg, respiratory rate of 28 breaths/min, and oxygen saturation of 90 % in room air, which improved to 96 % on 3 L/min supplemental oxygen administered intranasally. Pulmonary auscultation revealed a decreased breath sound over the right lung field. She had normal heart sounds without murmurs. A large, hard, non-tender mass was palpable in the lower part of the abdomen. There was no finger clubbing, cyanosis, lymphadenopathy, or edema in her extremities.

The complete blood count, blood biochemistry, and autoantibodies were all unremarkable, except for serum carcinoembryonic antigen (CEA) at 6.1 ng/mL (normal, below 5.0 ng/mL) and carbohydrate antigen 125 (CA-125) at 260 U/mL (normal, below 35 U/mL). The chest radiography showed cardiac enlargement and right pleural effusion, as well as blunting of the left costophrenic angle (Fig. 1c). Her electrocardiogram (ECG) was within normal limits. A transthoracic echocardiogram showed preserved right and left ventricular function with pericardial effusion. A chest, abdominal, and pelvic CT scan showed the presence of pericardial and bilateral pleural effusion and ascites with a left ovarian mass, 20 cm in diameter, without hepatosplenomegaly or lymphadenopathy (Fig. 1d). Pericardial effusion had decreased compared with that before the lung operation. A thoracentesis revealed clear and yellowish pleural fluid. It was a lymphocyte-predominant exudate; the total protein level in the pleural fluid was 3.1 g/dL (6.2 g/dL in serum) and lactate dehydrogenase (LDH) in pleural fluid was 181 U/L (238 U/L in serum), which fulfilled Light’s criteria for an exudate [8]. The adenosine deaminase (ADA) and CEA levels in the pleural fluid were 9.7 U/L and 2.4 ng/mL, respectively. The bacterial and mycobacterial cultures of the pleural fluid were negative. Cytological examination of the pleural fluid showed abundant lymphocytes without any evidence of malignant cells, similar to that in the pericardial effusion confirmed during the lung surgery.

**Fig. 3:**
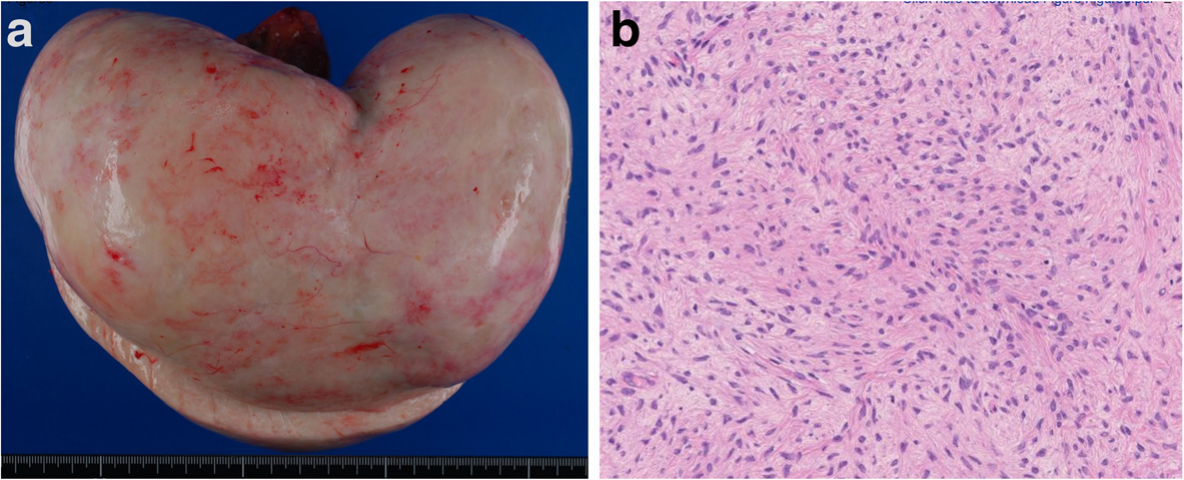
Pathologic findings of the ovarian fibroma. Macroscopic appearance showed a solid and smooth mass (**a**). Microscopic findings with hematoxylin and eosin staining showed spindle cells that were arranged in intersecting bundles (**b**)

Despite drainage of the pleural fluid, recurrent episodes of pleural fluid accumulation occurred requiring weekly thoracentesis. Despite tube thoracostomy into the right pleural cavity for drainage, the pleural effusion persisted. As the combination of her ascites, pleural effusion, and ovarian mass was suggestive of Meigs’ syndrome, she was referred to the department of gynecology and underwent bilateral salpingo-oophorectomy due to strong adhesions of bilateral ovaries. Histopathological examination of the resected ovarian mass revealed ovarian fibroma (Fig. 3). Thereafter, she made an uneventful recovery and the volume of pleural and pericardial effusions, as well as ascites, decreased and subsequently resolved. The follow-up chest X-ray and CT 8 months after removal of the fibroma confirmed no relapse of the pleural and pericardial effusion (Fig. 1e, f).

## Discussion

Meigs’ syndrome is defined as a condition that meets the following diagnostic criteria: the presence of pleural effusion and ascites; the existence of benign and solid ovarian tumors with the gross appearance of a fibroma (fibroma, thecoma, and granulose cell tumor); and the ascites and pleural effusion rapidly resolve after removal of the tumor [1]. In 1887, Demons initially reported nine cases with ovarian cysts that were cured of their ascites and hydrothorax by removal of the cyst [9]. Thereafter, in 1937, Meigs and Cass reported a series of seven patients who had ascites and pleural effusion associated with benign ovarian fibroma [3]. Meigs’ syndrome is more common in postmenopausal women with an average age of about 50 years [10]. Since the original description reported by Meigs, other benign ovarian tumors and some gynecological conditions associated with ascites and hydrothorax, such as degenerative ovarian changes and ovarian hyperstimulation syndrome, have been reported and referred to as pseudo-Meigs’ syndrome [11, 12].

Meigs’ syndrome with pericardial effusion is a very rare condition and has been termed Meigs’-like syndrome [5]. Of three cases in which Meigs’-like syndrome was suspected [5–7], ovarian fibroma was histopathologically confirmed in only one case [5]; the ovarian tumor was not resected in the other two cases. Considering that pericardial effusion was simultaneously detected with other findings such as pleural effusion and ascites in the three cases reported previously, the preceding pericardial effusion followed by pleural effusion in our case was a unique presentation.

The pathogenesis of the production of ascites and pleural effusion in Meigs’ syndrome is unknown. Past reports suggest that the ascitic fluid results from edematous fibromas that leak fluid or increased lymphangial pressure in the pelvis and abdomen caused by the tumor itself [1]. It has also been supposed that the pleural effusion arises secondary to the ascites through a congenital defect of the diaphragm or diaphragmatic lymphatics [1]. In addition, another theory is that substances such as VEGF that raise capillary permeability and some inflammatory cytokines including interleukin (IL)-1β, IL-6, and IL-8 are possible etiologies of ascites and hydrothorax formation [4]. VEGF is reportedly associated with the production of peritoneal and pleural fluid in patients with malignant and nonmalignant diseases [13]. The increase in VEGF levels in serum, peritoneal, and pleural fluid in Meigs’ syndrome may be attributable to VEGF production by the tumor [14]. Unfortunately, the VEGF level could not be measured in our case.

Although pleural fluid analysis plays an important role in diagnosing patients with pleural effusion, contradictory data regarding pleural fluid characteristics have been reported in patients with Meigs’ syndrome, as some studies have classified it as a transudate [15, 16], whereas others stated that it is an exudate [17, 18]. Pleural fluid is generally characterized as either a transudate or an exudate based on Light’s criteria, which assess serum and pleural fluid protein and LDH [8]. Transudative pleural effusion occurs in patients with imbalances in hydrostatic and oncotic pressures in the chest, such as chronic heart failure and nephrosis. In contrast, exudative pleural effusion results from a variety of mechanisms, including infection, malignancy, and trauma. According to a systematic review of 653 studies on Meigs’ syndrome, the majority of pleural effusions in patients are exudates, as is in our case, despite limited data reliably defining the characteristics of the pleural effusion [19]. In addition, we needed to carefully differentiate Meigs’ syndrome from tuberculous pleural effusion, because the pleural effusion in our case was a lymphocyte-predominant exudate consistent with tuberculous pleurisy. As acid-fast bacilli stains and cultures are of low yield in the setting of tuberculous pleural effusion, such negative bacterial findings cannot rule out tuberculous pleurisy [20]. When the bacterial evaluation of pleural fluid is not diagnostic, pleural biopsy, culture of pleural tissue, and an elevated pleural fluid ADA level are often useful to confirm the diagnosis [21]. Pleural effusion in our case was unlikely to be caused by tuberculosis, due to the normal ADA level and the negative mycobacterial culture findings.

High levels of serum CA-125 are suggestive of ovarian malignancy, and levels have been reported to increase in Meigs’ syndrome [22]. This antigen is produced by many clinical tissues, including the epithelium of fallopian tubes, endometrium, and mesothelium of the pleura, pericardium, and peritoneum [23]. In a previous report, immunohistochemical staining for CA-125 confirmed that the serum elevation of CA-125 in a patient with Meigs’ syndrome was correlated with mesothelial expression of the antigen rather than the fibroma [24]. Therefore, careful interpretation is needed in the case of elevated serum CA-125 levels whenever serosal (peritoneum, pleura, and pericardial) fluid is present as in Meigs’ syndrome [25].

Pericardial effusion can occur in a large variety of underlying diseases. A review of 173 consecutive patients undergoing pericardiocentesis reported that the most common cause was malignancy (33 %), followed by chronic-idiopathic causes (14 %) [26]. Another study revealed that the etiology of pericarditis associated with pericardial effusion was not defined in up to 30 % of patients, despite the progression of diagnostic techniques such as pericardioscopy and immunohistochemistry [27]. The common ECG findings in patients with a large volume of pericardial effusion are sinus tachycardia, low QRS voltage, and electrical alternans [28], which were not seen in our case at admission or before the lung surgery. Pericardial drainage is indicated for tamponade, purulent effusion, or for recurrent or large idiopathic effusions with hemodynamic compromise or suspicion of neoplastic or tuberculous causes [29]. Cardiac tamponade is a life-threatening entity requiring prompt drainage of pericardial fluid and can occur when a large volume of pericardial fluid acutely or subacutely accumulates. In contrast, a slow accumulation of pericardial effusion over several weeks does not increase pericardial pressure sufficiently to cause symptomatic cardiac tamponade, despite that the volume of pericardial effusion can be measured in liters [30]. Although surgical approaches including pericardial fenestration and pericardiotomy should be considered in cases with recurrent large effusion, postoperative pleural effusion that has moved from the pericardial space through a pericardial window rarely becomes a problem [31]. In our case, pleural effusion increased after surgery for localized lung cancer with pericardial fenestration, in contrast to decreasing pericardial effusion. We speculate that pericardial effusion, the volume of which had not changed for more than 3 years due to a closed pericardial space maintaining a well-balanced pericardial pressure, overflowed into the thoracic cavity through a pericardial window and accumulated as pleural effusion. Similar cytological findings, including abundant lymphocytes between the peripheral and pleural fluid, strengthen our hypothesis.

It is of great interest that pericardial effusion preceded pleural effusion in our case, and not only pleural effusion and ascites but also pericardial effusion disappeared without reaccumulation after the removal of the ovarian tumor. Any other possible causes of pericardial effusion, such as infectious pericarditis, malignancy, and connective tissue disease, were not plausible in our case, although a long-term follow-up is necessary to detect signs of lung cancer recurrence. Therefore, we considered that the pericardial effusion was associated with the ovarian fibroma. The presence of preceding pericardial effusion cannot be explained by a common hypothesis suggesting that hydrothorax is secondary to ascites in Meigs’ syndrome [1], as it is difficult for ascites to move directly into the pericardial space via the peritoneal cavity. Preceding pericardial effusion may imply that substances such as inflammatory cytokines and hormonal stimulation caused by the tumor play an important role in the accumulation of fluid in Meigs’ syndrome. Interestingly, Mertin et al. reported an atypical case of Meigs’ syndrome with bilateral pleural effusion and without ascites in a granulosa cell tumor [32].

The most appropriate treatment for Meigs’ syndrome is removal of the tumor. The pleural effusion and ascites resolve within a few weeks after tumor resection. A unilateral salpingo-oophorectomy is commonly performed for the treatment of an ovarian tumor. For women who desire preservation of the ovary, an ovarian cystectomy may be performed with complete excision of the fibromatous tissue [33]. The prognosis of Meigs’ syndrome is favorable and recurrence of peritoneal and pleural fluid after complete removal of the tumor is unlikely to occur.

## Conclusion

To the best of our knowledge, this is the first case of Meigs’ syndrome with preceding pericardial effusion in advance of pleural effusion. It is important for the clinician to consider the possibility of Meigs’ syndrome in female patients with unexplained pericardial effusion and an ovarian tumor. Although a malignant disease should be suspected in all patients with undiagnosed pleural and/or pericardial effusion, Meigs’ syndrome is a disease curable by tumor resection and should be differentiated from malignancy.

## Consent

Written informed consent was obtained from the patient to publish this case report and any accompanying images. A copy of the written consent is available for review by the Editor-in-Chief of this journal.

## Availability of data

The datasets supporting the conclusions of this article are included within the article.

## Abbreviations

ADA: adenosine deaminase
CA-125: carbohydrate antigen 125
CEA: carcinoembryonic antigen
CT: computed tomography
ECG: electrocardiogram
IL: interleukin
LDH: lactate dehydrogenase
VEGF: vascular endothelial growth factor

## References

[1] Meigs JV (1954). Fibroma of the ovary with ascites and hydrothorax; Meigs’ syndrome. Am J Obstet Gynecol.

[2] Chechia A, Attia L, Temime RB, Makhlouf T, Koubaa A (2008). Incidence, clinical analysis, and management of ovarian fibromas and fibrothecomas. Am J Obstet Gynecol.

[3] Meigs JV, Cass JW (1937). Fibroma of the ovary with ascites and hydrothorax. With a report of seven cases. Am J Obstet Gynecol.

[4] Abramov Y, Anteby SO, Fasouliotis SJ, Barak V (2002). The role of inflammatory cytokines in Meigs’ syndrome. Obstet Gynecol.

[5] Qaisar S, Osman F, Pitt M (2005). Resolution of pericardial effusion after removal of ovarian fibroma— a Meigs’-like syndrome. J R Soc Med.

[6] Patane S, Marte F, Di Bella G, Davi M (2008). Pericardial effusion with elevated serum carbohydrate antigen 125 levels and ovarian tumor mass. Int J Cardiol.

[7] Arnaiz-Garcia ME, Gonzalez-Santos JM, Lopez-Rodriguez J, Dalmau-Sorli MJ, Bueno-Codoner ME, Arnaiz J (2013). Meigs-like syndrome presenting as cardiac tamponade. Revista portuguesa de cardiologia : orgao oficial da Sociedade Portuguesa de Cardiologia = Portuguese Journal of Cardiology : an official journal of the Portuguese Society of. Cardiology.

[8] Light RW, Macgregor MI, Luchsinger PC, Ball WC (1972). Pleural effusions: the diagnostic separation of transudates and exudates. Ann Intern Med.

[9] Brun J-L (2007). Demons syndrome revisited: A review of the literature. Gynecol Oncol.

[10] Shiau CS, Chang MY, Hsieh CC, Hsieh TT, Chiang CH (2005). Meigs’ syndrome in a young woman with a normal serum CA-125 level. Chang Gung Med J.

[11] Morell ND, Frost D, Ziel HK (1980). Pseudo Meigs’ syndrome. A case report. J Reprod Med.

[12] Hardy E (1987). Meigs’ syndrome and pseudo-Meigs’ syndrome. J R Soc Med.

[13] Kraft A, Weindel K, Ochs A, Marth C, Zmija J, Schumacher P, Unger C, Marme D, Gastl G (1999). Vascular endothelial growth factor in the sera and effusions of patients with malignant and nonmalignant disease. Cancer.

[14] Ishiko O, Yoshida H, Sumi T, Hirai K, Ogita S (2001). Vascular endothelial growth factor levels in pleural and peritoneal fluid in Meigs’ syndrome. Eur J Obstet Gynecol Reprod Biol.

[15] Hooper C, Lee YC, Maskell N (2010). Investigation of a unilateral pleural effusion in adults: British Thoracic Society Pleural Disease Guideline 2010. Thorax.

[16] McGrath EE, Blades Z, Needham J, Anderson PB (2009). A systematic approach to the investigation and diagnosis of a unilateral pleural effusion. Int J Clin Pract.

[17] Agaba EI, Ekwempu CC, Ugoya SO, Echejoh GO (2007). Meigs’ syndrome presenting as haemorrhagic pleural effusion. West Afr J Med.

[18] Riker D, Goba D (2013). Ovarian mass, pleural effusion, and ascites: revisiting Meigs syndrome. J Bronchology Int Pulm.

[19] Krenke R, Maskey-Warzechowska M, Korczynski P, Zielinska-Krawczyk M, Klimiuk J, Chazan R, Light RW (2015). Pleural effusion in meigs’ syndrome-transudate or exudate?: Systematic review of the literature. Medicine.

[20] Seibert AF, Haynes J, Middleton R, Bass JB (1991). Tuberculous pleural effusion. Twenty-year experience. Chest.

[21] Gopi A, Madhavan SM, Sharma SK, Sahn SA (2007). Diagnosis and treatment of tuberculous pleural effusion in 2006. Chest.

[22] Benjapibal M, Sangkarat S, Laiwejpithaya S, Viriyapak B, Chaopotong P, Jaishuen A (2009). Meigs’ Syndrome with elevated serum CA125: Case report and review of the literature. Case Rep Oncol.

[23] Kabawat SE, Bast RC, Bhan AK, Welch WR, Knapp RC, Colvin RB (1983). Tissue distribution of a coelomic-epithelium-related antigen recognized by the monoclonal antibody OC125. Int J Gynecol Pathol.

[24] Timmerman D, Moerman P, Vergote I (1995). Meigs’ syndrome with elevated serum CA 125 levels: two case reports and review of the literature. Gynecol Oncol.

[25] Sevinc A, Camci C, Turk HM, Buyukberber S (2003). How to interpret serum CA 125 levels in patients with serosal involvement? A clinical dilemma. Oncology.

[26] Ben-Horin S, Bank I, Guetta V, Livneh A (2006). Large symptomatic pericardial effusion as the presentation of unrecognized cancer: a study in 173 consecutive patients undergoing pericardiocentesis. Medicine.

[27] Maisch B, Ristic AD (2002). The classification of pericardial disease in the age of modern medicine. Curr Cardiol Rep.

[28] Spodick DH (2003). Acute cardiac tamponade. N Engl J Med.

[29] Soler-Soler J, Sagrista-Sauleda J, Permanyer-Miralda G (2001). Management of pericardial effusion. Heart.

[30] Shabetai R (2004). Pericardial effusion: haemodynamic spectrum. Heart.

[31] Sagrista-Sauleda J, Angel J, Permanyer-Miralda G, Soler-Soler J (1999). Long-term follow-up of idiopathic chronic pericardial effusion. N Engl J Med.

[32] Martin F, Brouche S, Haidar A (1990). Demons-Meigs’ syndrome. Report of a case with ovarian tumor of the granulosa. Rev Pneumol Clin.

[33] Cho YJ, Lee HS, Kim JM, Lee SY, Song T, Seong SJ, Kim ML (2015). Ovarian-sparing local mass excision for ovarian fibroma/fibrothecoma in premenopausal women. Eur J Obstet Gynecol Reprod Biol.

